# Sponge cities for healthier populations

**DOI:** 10.2471/BLT.23.020223

**Published:** 2023-02-01

## Abstract

Nature-based approaches to enhancing the absorbency of urban environments are being used to address the linked issues of climate change, flooding, sanitation and health. Fid Thompson reports.

Yu Kongjian’s understanding of flood management developed when he was growing up in rural China. “I lived in a village in Zhejiang Province, and we had seven ponds which we used to regulate the monsoon flood,” recalls the 60-year-old. Architect Yu started designing buildings that incorporated permeable surfaces, and the idea of the sponge city (*Hăimián chéngshì*) was born.

The sponge city, as the name suggests, incorporates absorbent elements such as wetlands, ponds, rice paddies, and parks in floodplain areas. Concrete walls channelling river water are replaced with natural earthwork banks covered with vegetation and roads are lined with bioswales (water channels that also filter debris and pollutants).

“These elements allow rainwater to spread out or filter down into the earth, slowing down the potentially destructive flow of water in heavy storms,” explains Yu, now based in Beijing.

It was after severe flooding hit Beijing in July 2012, killing 79 people, that the Chinese authorities started to take a keen interest in Yu’s work, funding sponge city pilot projects which were launched in 2014 in two densely populated cities on the island of Hainan.

In the face of increasingly frequent extreme flooding events, such as the catastrophic flooding that hit Pakistan in 2022 caused by heavier-than-usual monsoon rains and run-off from melting glaciers, governments worldwide are taking a similar interest in absorbency and the way their infrastructure holds up when the rain comes down.

In Pakistan, infrastructure failure was often part of rapidly evolving crises. Raheema Panhwar, Provincial Coordinator for Sindh at international nongovernmental organization WaterAid, was helping people affected by the flooding in Badin District, Sindh province, when she heard about the breaching of the Mirpurkhas Main Drain which took place on 28 August 2022.

“I drove with my colleagues to the affected area and found that hundreds of villages had been inundated,” she says. “People had lost everything, and we spent the next couple of days traversing the Dadu, Badin and Jamshoro Districts bringing water, jerry cans, temporary toilets and hygiene kits to women and young girls.”

For Karen Sudmeier, an expert in nature-based solutions for disaster risk reduction who has a long association with the United Nations Environmental Programme (UNEP), the breaching of the Mirpurkhas Main Drain is emblematic of flood-related infrastructure failures that are occurring with increasing frequency worldwide.

“We are confronting the limitations of the materials and the structures we use,” she says. “Non-porous, non-absorbent materials, such as concrete and asphalt, and inflexible structures such as pipes, dams and drains just aren’t adapted for extreme flooding events. They can’t absorb water, so they channel or pool it; they can’t bend, so they break.”

Cities are full of such materials and it is therefore no surprise that they often bear the brunt of flooding, as was the case in Beijing in 2012 or more recently in Kinshasa in the Democratic Republic of the Congo, which was hit by flooding in mid-December 2022 causing over 140 deaths.

“We are confronting the limitations of the materials and the structures we use.”

Karen Sudmeier

Such events constitute significant public-health emergencies, entailing increased exposure to injury, drowning and – where sanitation systems are inadequate – outbreaks of infectious disease.

“The people most affected by floods and the health risks they entail are those living in informal settlements located in urban floodplains, without adequate water and sanitation systems or services,” explains Kate Medlicott, the Sanitation and Waste team leader within the water, sanitation and hygiene (WASH) unit at the World Health Organization (WHO).

According to Medlicott, some 1.7 billion people, around 38% of all urban dwellers, currently lack safe sanitation systems, using a pit toilet or septic tank at home rather than being connected to a sewer system. In flooding events, such systems are the first to be overwhelmed.

“Makeshift pit toilets, septic tanks and combined sewer and stormwater are more likely to overflow leaving low-lying areas of the city awash with human excreta,” Medlicott explains. “Increased outbreaks of waterborne diseases, including cholera, are a natural consequence.”

In an attempt to address these interrelated problems, like Yu Kongjian, an increasing number of urban planners and architects are introducing “green infrastructure” elements into the fabric of cities.

Part of a broader panoply of what are referred to as nature-based solutions (initiatives relying on the exploitation of natural features and mechanisms to tackle socio-environmental challenges), green infrastructure includes constructed wetlands, parks, green roofs, rain gardens, green sidewalks, permeable pavements, and bioswales.

According to Sudmeier, green infrastructure projects are taking root worldwide, ranging from the projects being implemented across China, to smaller, focused projects such as the “green corridor” which was opened along a disused railway line in Singapore in July 2022, or the riverside park introduced in Beira, Mozambique, as part of a combined green/grey infrastructure project designed to mitigate flooding.

The focus on infrastructure that underpins sanitation is bringing it into discussions of climate-change-impact mitigation. Not a moment too soon as far as Juliet Willetts, Research Director at the University of Technology Sydney, is concerned. “There’s a large gap in awareness,” she says, “and sanitation has been completely absent from climate-related documents.”

According to Willetts, only a handful of countries have mentioned sanitation in their national climate change adaptation plans.

Indonesia is one of them. Frequently subject to flooding, Indonesia’s densely populated cities suffer from poor drainage and sanitation systems, most people using unsealed and untreated, individual septic tanks.

The vulnerabilities of such arrangements were highlighted in a 2021 study of four cities carried out by the Government of Indonesia and the United Nations Children’s Fund (UNICEF). “The study really raised awareness that climate change impacts sanitation as well as water supply,” says Maraita Listyasari, an urban development specialist at UNICEF Indonesia. “We studied water sources for 20 000 households and found that almost 80% were contaminated by faecal waste, a finding that was widely reported and prompted significant action on the part of the government.”

“Sanitation has been completely absent from climate-related documents.”

Juliet Willetts

That action includes the testing of several innovative approaches to creating climate-resilient sanitation systems, including improving drainage and sanitation by protecting natural springs and building infiltration ponds to absorb water overflow across 14 cities.

Willetts applauds such initiatives but would like to see more. “We really need to see the political will and greater investment,” she says.

According to Sudmeier, the cost of implementing nature-based solutions is often cited as an obstacle to pursuing them, but she questions the evidence for such arguments. “Green infrastructure has initial costs, as does grey infrastructure, but it has lower maintenance costs and brings so many additional benefits, including reducing heat in cities, that over the long term it saves money,” she argues, citing a cost/benefit and equity analysis of upgrading New York City’s infrastructure conducted by UNEP in 2010 which indicated that green infrastructure was cheaper over a 10-year period.

The intersectoral nature of the climate/sanitation issue is another challenge often cited. “Sanitation tends to be the province of one ministry but needs to be seen as a multisectoral challenge,” says Willetts. “As such, it requires multisectoral responses and policies, with climate risks considered in sanitation plans and sanitation concerns addressed in climate resilience planning.”

Here too Indonesia is showing the way, by beginning to mainstream climate adaptation and mitigation in health and WASH sector planning. In 2019, the health ministry developed a National Action Plan on Adaptation to Climate Change in the Health Sector with WHO, including technical guidelines for building climate resilience into the health sector at the local level.

Medlicott points out that since 2018, WHO has also integrated the assessment and management of climate risks into all sanitation guidelines and tools and takes every opportunity to promote the climate-resilient sanitation agenda. “At last year’s COP27 there was a first-ever dedicated session on sanitation at the water pavilion, where we launched a call to action on climate-resilient sanitation and, for the first time, the outcome document recognized the critical role of protecting, conserving and restoring water systems and water-related ecosystems, opening the way to more focus on sanitation and drainage in climate finance and plans,” she says.

Sudmeier hopes that this sharpened focus on water-related ecosystems will encourage policy-makers and urban planners to consider nature-based solutions as part of their city development projects. “One of the biggest challenges we face is making sure that the cities we build and/or expand are safe, resilient and sustainable,” she says. “By embracing nature-based solutions, urban planners can weave climate resilience into their very fabric while also mitigating climate-related health risks.”

In China, Yu is already working on it. “Since 2014, the government has extended its support, and is currently backing 56 sponge city projects across China,” he says. “The plan is to reach 80% of urban areas with green sponge elements by 2030.” There is clearly a lot of work to do.

**Figure:**
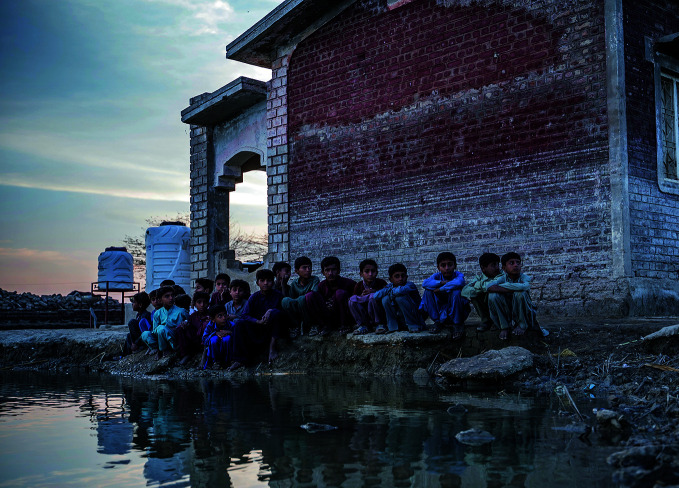
Children beside contaminated flood water near a village in Dadu District, Sindh.

UNICEF/Arsalan Butt

**Figure:**
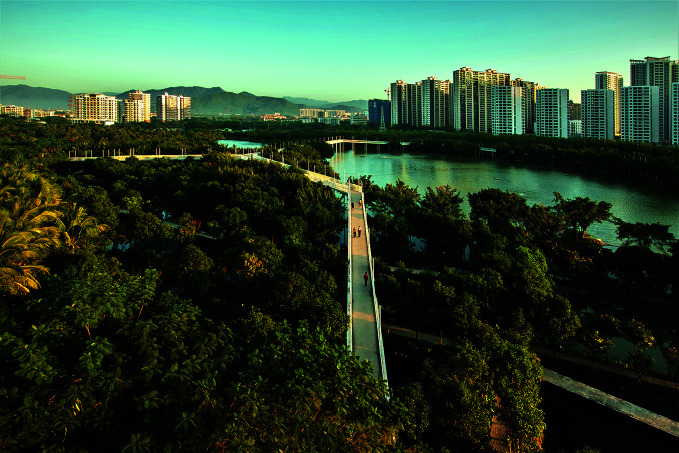
Dong'an Wetland Sponge City Project, Hainan, China.

Courtesy of Yu Kongjian

